# Stereotypy and variability of social calls among clustering female big-footed myotis (*Myotis macrodactylus*)

**DOI:** 10.24272/j.issn.2095-8137.2018.026

**Published:** 2018-03-07

**Authors:** Yan-Hong Xiao, Lei Wang, Joseph R. Hoyt, Ting-Lei Jiang, Ai-Qing Lin, Jiang Feng

**Affiliations:** 1 Jilin Provincial Key Laboratory of Animal Resource Conservation and Utilization, Northeast Normal University, Changchun Jilin 130117, China; 2 School of Animal Science & Technology, Jilin Agricultural University, Changchun Jilin 130118, China; 3 Department of Ecology and Evolutionary Biology, University of California, Santa Cruz CA 95064, USA

**Keywords:** Social calls, Clustering, Big-footed myotis

## Abstract

Echolocating bats have developed advanced auditory perception systems, predominantly using acoustic signaling to communicate with each other. They can emit a diverse range of social calls in complex behavioral contexts. This study examined the vocal repertoire of five pregnant big-footed myotis bats (*Myotis macrodactylus*). In the process of clustering, the last individual to return to the colony (LI) emitted social calls that correlated with behavior, as recorded on a PC-based digital recorder. These last individuals could emit 10 simple monosyllabic and 27 complex multisyllabic types of calls, constituting four types of syllables. The social calls were composed of highly stereotyped syllables, hierarchically organized by a common set of syllables. However, intra-specific variation was also found in the number of syllables, syllable order and patterns of syllable repetition across call renditions. Data were obtained to characterize the significant individual differences that existed in the maximum frequency and duration of calls. Time taken to return to the roost was negatively associated with the diversity of social calls. Our findings indicate that variability in social calls may be an effective strategy taken by individuals during reintegration into clusters of female *M. macrodactylus*.

## INTRODUCTION

Animals have evolved a variety of communication systems across all sensory modalities, such as visual, chemical, electrical, and acoustic signals. Of the communication modalities, acoustic signals have the advantage of broadcasting information for longer distances with less interference from physical barriers. This advantage was likely a driving force in the evolution of acoustic communication in a wide range of taxa (Seyfarth et al., 2010; Wilkins et al., 2012). Acoustic communication fulfills an important function in social interactions, especially for bats, who are among the most diverse and gregarious of all mammals and live in predominately dark environments (Kerth, 2008). The acoustic communication systems observed in echolocating bats are extremely complex in the mammalian world because they can emit two kinds of calls: echolocation and social calls, which serve different functions (Kanwal, 2009, 2012). Social calls are mainly used to transmit information under special behavioral contexts. Many bat species demonstrate a diverse repertoire of social calls under a variety of behavioral conditions (Bohn et al., 2008; Clement et al., 2006; Gadziola et al., 2012; Knörnschild et al., 2010; Ma et al., 2006). These characteristics of bats provide a unique opportunity for research on acoustic communication of animal sociality.

The motivation-structure hypothesis states that social call types are decided by the behavioral context of birds and mammals (Kanwal, 2009; Morton, 1977). In bats, the behavioral settings of their vocalizations include aggressive or warning, courtship, communication, distress, mother-infant recognition, and clustering behaviors. Intra-specific communication under specific behavioral contexts usually carries significant information about the signaler (Behr et al., 2009; Behr & von Helversen, 2004; Knörnschild and von Helversen, 2008). Under some behaviors, intra-specific differences and elaborate sequences of acoustic signals are of importance, and contribute to accurate mother-infant and individual recognition within a colony. For instance, songs of free-tail bats contain multiple elements (e.g., syllables, notes and/or phrases) with highly structured, rather than random, arrangement. Males often modify types of calls through modulating the repeated syllables, which can differ among individuals (Bohn et al., 2009). Clustering behavior is another important context in which bats emit social calls. Mammals living in low-temperature ambient environments often huddle together to reduce energy consumption and promote member fitness (Gilbert et al., 2010). The central temperature of a cluster is higher, and the central site is safer than the border position (Hatchwell et al., 2009). Thus, achieving the central position is often the objective of an individual within a cluster. Acoustic signals containing characteristic information may contribute to accurate recognition, which can help individuals save energy and time during contention for the central cluster site (Dugatkin and Earley, 2004). Although several studies have examined the clustering behavior of bat species (Kerth, 2008), there have been no studies on the diversity and structural characteristics of acoustic communication that occur during the clustering of gregarious bats.

Big-footed myotis, *Myotis macrodactylus*, belongs to the subgenus *Leuconoë* of the genus *Myotis* (Yangochiroptera, Vespertilionidae). Sexual segregation in colonies is known to occur in many bat species, with separate clustering of females during the summer pupping season (Bradbury, 1977; McCracken and Wilkinson, 2000). Females rear their young communally with the formation of maternity colonies, whereas males are solitary or form small bachelor groups (Senior et al., 2005). The clustering of pregnant bats is a cooperative behavior that permits individuals involved in social thermoregulation to minimize heat loss and thereby lower their energy expenditure, and may allow them to reallocate the saved energy to other functions such as growth and reproduction (Gilbert et al., 2010). Thus, the cluster center is the optimal position for pregnant bats, and may assist better fetal development. According to our observations, the last individuals (LI) returning to a group will usually try to enter the central position of the cluster. Approaching behaviors are always accompanied by social calls produced by the LI until physical contact with the other bats. Thus, we assumed that the social calls emitted by the LI attempting to enter the cluster center are related to this special behavior. We therefore examined two questions: (1) Are these social calls associated with successful entrance into the center of the cluster? (2) Does the variety or structure of the social calls influence this behavior? We recorded and analyzed the social calls emitted by *M*. *macrodactylus* under this behavioral context. 

## MATERIALS AND METHODS

### Acquisition and maintenance of animals

The study was conducted from May to June 2010. Five pregnant *M*. *macrodactylus* individuals were randomly chosen from 90 pregnant bats in a natural cave in Jilin Province, China. They were banded and roosted together as a colony in a temperature- and humidity-controlled animal room (20 m×10 m×4 m) at Northeast Normal University in Changchun, China. The day-night cycle was controlled at 12 h dark and 12 h light. They could fly freely within the animal facility and were given water and mealworms *ad libitum*. Before the experiment, all five subjects were habituated to handling and to the testing cage.

### Acoustic recordings

We recorded and analyzed the social calls and associated behaviors of the captive bats. To optimize recording quality, the five animals were housed in a small testing cage (200 cm×80 cm×100 cm) in a room lined with anechoic foam, separate from the animal holding room. To reduce environmental interference with the ultrasonic calls, environmental temperature and humidity of the room were kept constant. 

**Figure 1:**
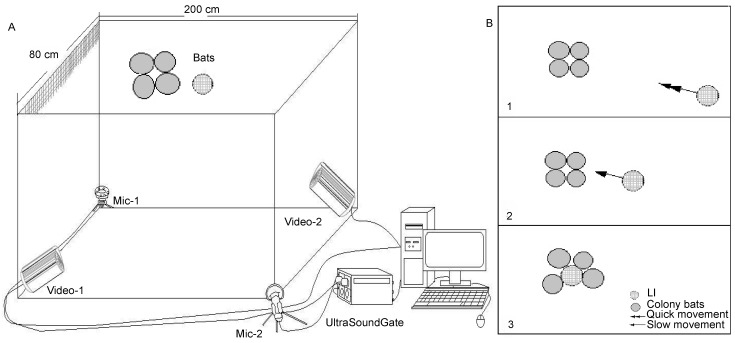
Schematic showing the cage set-up for simultaneous audio and video recordings of captive *M. macrodactylus*

Vocalizations were recorded using two ultrasonic condenser microphones (CM16, Avisoft Bioacoustics, Berlin, Germany) connected to amplifiers and A/D converters (UltraSoundGate 416 H). The microphone had a flat frequency response from 10–250 kHz. The gain of each microphone was independently adjusted to optimize the signal-to-noise ratio of the recording. Acoustic signals were digitized at 375 kHz with 16-bit depth, and monitored in real time with RECORDER software. We used two infrared surveillance cameras (Pensee, PIS-322EG) to simultaneously record the behavior of all animals. We defined the last animal to join the group (LI) as the last individual moving toward the cluster and displaying “approaching” behavior (Kobayasi et al., 2012). In most cases, the LI was observed as roosting last within a cluster. Most recordings occurred during the clustering process after bats had eaten (Figure 1). The characteristics of their social calls and the time they took to join the cluster were analyzed using Avisoft-SasLab Pro software, Version 5.1.20 (Avisoft Bioacoustics, Berlin, Germany). Time here was defined as consumed time (CT). The study was conducted for 38 d from 2 100 h–0 100 h each night. 

A: Testing was conducted in a wire mesh cage (200 cm×80 cm×100 cm). Two ultrasonic microphones (UltraSoundGate CM16) were placed outside the cage and pointed at the center of the ceiling of the cage, which was about 50 cm above the tip of the microphone. Two video monitors were used to monitor and record behaviors. B: Cluster of bats (illustrated as solid circles) and approaching bat (dotted circle), both in a bottom-up view. The sketch, taken from a sequence of video frames, shows the LI approaching the colony after foraging.

### Analysis of vocalizations

**Figure 2:**
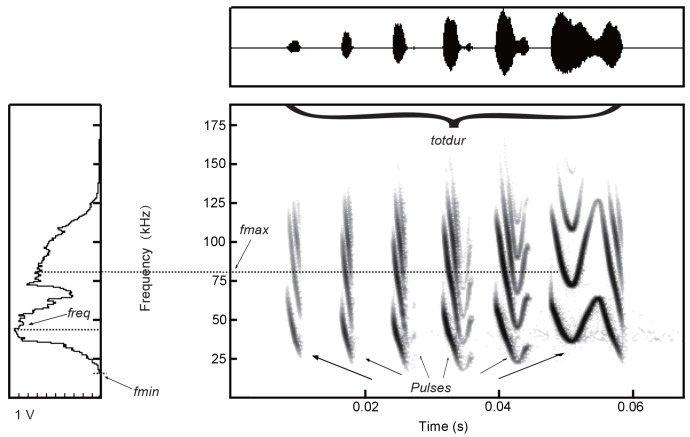
Spectrograms (center) of social calls of *Myotis macrodactylus* showing the change of frequency with time

We examined the social call sequences of the LI using nomenclature similar to Kanwal et al. (1994) and Bohn et al. (2008), with a syllable considered the smallest acoustic unit of a vocalization defined as one continuous emission surrounded by background noise, and a call considered the simplest emission pattern of a vocalization used for communication consisting of one or more elements (syllables). Vocalizations were semi-automatically analyzed using Avisoft-SasLab Pro software (Version 5.1.20 Avisoft Bioacoustics). Syllable start and end times were automatically detected using a standard threshold level (5%) and hold time (2 ms), and manually adjusted when necessary. Signals were high-pass filtered at 1 kHz and the amplitude of each pulse was normalized to 0.75 V (Bohn et al., 2008; Gadziola et al., 2012; Kanwal et al., 1994). As a result, social call sequences were of similar amplitude and unwanted echolocation signals were removed. For analyses, we randomly selected three calls per call type from all individuals that were of sufficient quality for measurements and syllable identification. The social calls were automatically analyzed with the Avisoft-SasLab Pro software (Version 5.1.20 Avisoft Bioacoustics). Spectral and temporal analyses were obtained from the spectrogram and oscillogram with a Fast Fourier Transform (FFT) length of 512 points (87.5% overlap), which allowed for a frequency resolution of 732 Hz and temporal resolution of 0.171 ms. We measured the following parameters for each social call (Figure 2): total duration of call (*totdur*), minimum (*fmin*) and maximum (*fmax*) frequencies of call, and mean frequency of highest energy (*freq*). This was done by measuring the peak frequency of each component (and then calculating average); number of components to call (*nocomp*); and number of different syllables (*Difptype*) in each call (Russ et al., 2004; Russo & Jones, 1999). If more than one harmonic was present, values of the first (fundamental) harmonic were taken. Additionally, we used oscillograms and spectrograms to identify syllables within calls. We also examined the composition of each call in terms of the total number of each syllable type and the proportion of calls with syllables. Finally, for each call recorded, we assigned a call variant and call type based on its sequence of syllables.

Oscillogram (top) gives the change of amplitude with time. Mean frequencies (left part of spectrum) are the average amplitude of different frequencies of the measured time window. Parameters evaluated in this study are shown.

### Statistical analyses

Call parameters were checked for normality using the Kolmogorov-Smirnov test. Since all call parameters were normally distributed, differences in call characteristics between individuals were compared using one-way ANOVA, followed by Tukey multiple-comparison tests. Discriminant function analysis (DFA) was used to determine to which individual certain social calls should belong. We examined the general relationships between the variables across all the LIs, including call variants and CTs, by performing Spearman correlations. We used a simple linear regression analysis to assess the statistical significance of call variation as a predictor of individual recognition of CT in the process of approaching clusters. All statistical analyses were performed using SPSS 15.0 statistical software (SPSS Inc., Chicago, IL, USA). The alpha level was set to 0.05 and data were presented as means±*SD*. 

## RESULTS

### Acoustic structure of calls

**Figure 3:**
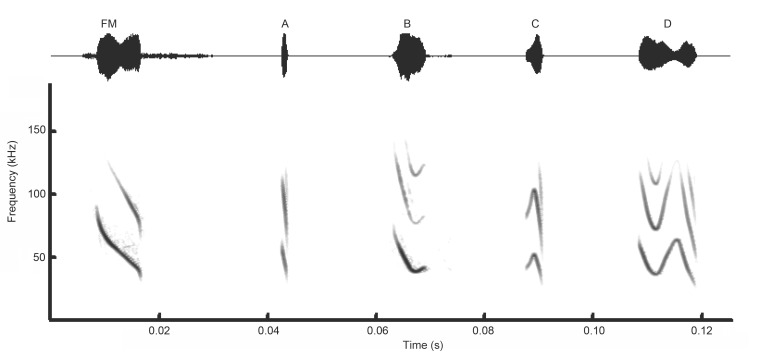
Echolocation pulse (FM) and simple syllables

**Figure 4:**
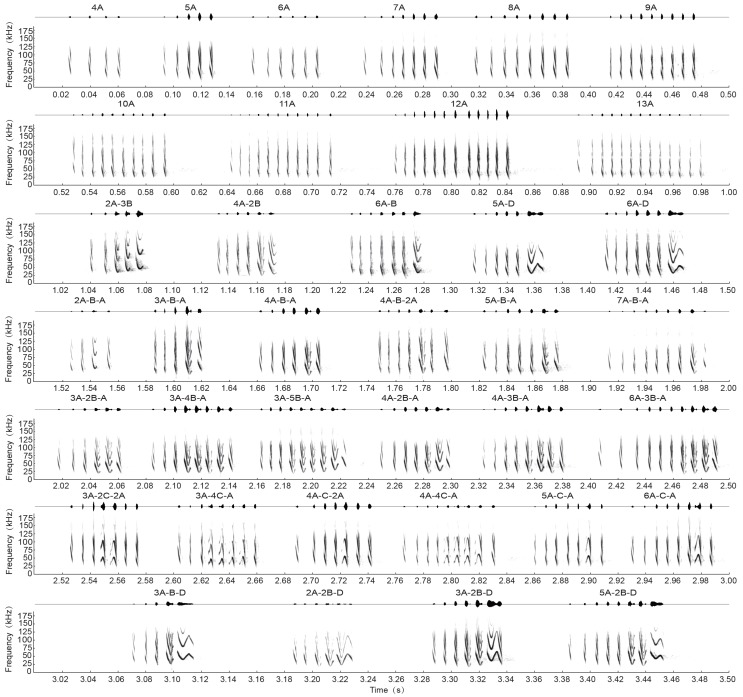
Spectrograms and oscillograms of multi-element social calls from female *Myotis macrodactylus*

**Figure 5:**
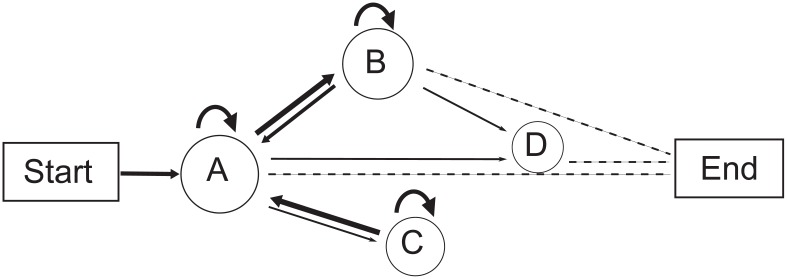
Model of calls based on transition syllables

A total of 885 call sequences were analyzed from five individuals when the LI approached the cluster. We distinguished a total of four syllable types, which could be combined to form 10 simple monosyllabic calls and 27 complex multisyllabic calls. The four syllable types and their 37 combinations are outlined in Figure 3 and Figure 4: downward frequency-modulated syllable (DFM; Figure 3A), short quasi-V frequency-modulated syllable (VFM; Figure 3B), arched frequency-modulated syllable (AFM; Figure 3C) and sinusoidal frequency-modulated syllable (SFM; Figure 3D). In the syllable sequence (Figure 5), social calls started with DFM syllables, which, in turn, usually gave way to VFM syllables. The VFM syllables were followed by SFM syllables, usually at the end of a call. The AFM syllables were only interjacent in the sequence and only made transition with DFM syllables. Typically, SFM syllables had a longer duration (10.4±2.8 ms) than that of the other syllable types (DFM, 1.6±0.7 ms; VFM, 4.2±0.8 ms; AFM, 3.5±0.8 ms). 

Oscillogram (top) of the syllable(s) with the corresponding spectrogram (bottom). Syllables are: A, downward frequency-modulated syllable (DFM); B, short quasi-V frequency-modulated syllable (VFM); C, arched frequency-modulated syllable (AFM); D, sinusoidal frequency-modulated syllable (SFM).

Arrows represent transitions from one syllable to the next. Arrow thickness increases with transition frequencies that deviate from the expected by more than 10%, 30%, or 50%. Dotted lines represent the end of a call. See Figure 4 for examples of each call.

### Individual acoustical differences

**Figure 6:**
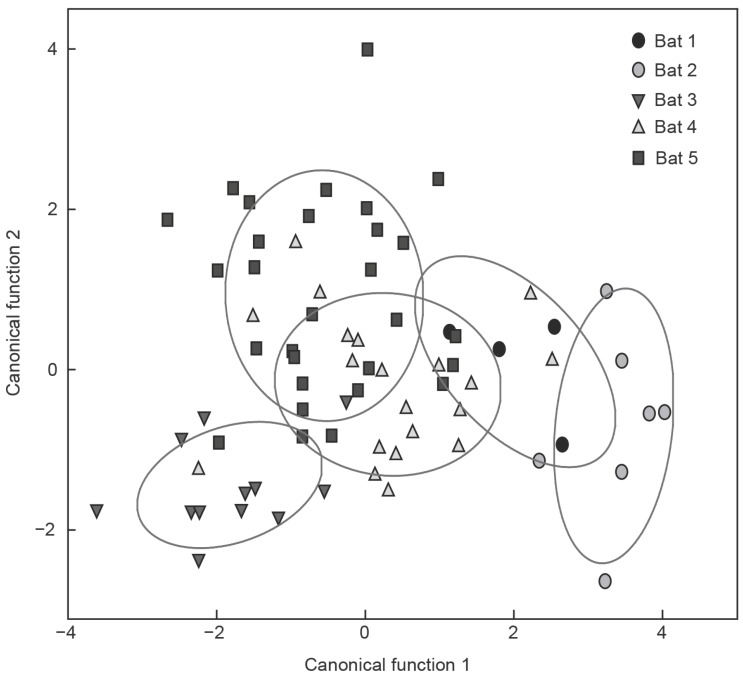
Distribution of social calls from five females (marked by different symbols) on first two canonical axes

To establish whether the social calls were individual-specific, we performed a DFA using four spectrotemporal and two syllable parameters (Table 1). This analysis resulted in two canonical functions with eigenvalues greater than 1, which together explained 90.1% of the variation in the calls from the five bats (Figure 6). Subsequent univariate ANOVA showed that the first and second canonical functions could be used to differentiate between social calls of individual bats (PC1: F_4, 67_=36.6, *P*<0.001; PC2: F_4, 67_=13.4, *P*<0.001). Maximum frequency and duration were the most distinctive features of these calls, indicating different acoustic repertoires. These analyses suggested that social calls from different individuals should be distinguishable by conspecifics.

**Table 1:** Social call parameters from *Myotis macrodactylus* females

Bat	Call (*n*)	Variants	*totdur* (ms)	*freq* (kHz)	*fmax* (kHz)	*fmin* (kHz)	*Pulses* (per 0.1s)	*Difptype*
1	33 (3)	4	46.6±10.5 b	49.4±5.2 a	100.4±6.7 a	23.6±3.1 b	5.5±1.2 a	1.0±0.0 a
2	51 (4)	7	37.8±7.9 a	49.6±9.3 a	99.1±8.5 a	25.5±3.6 a	4.9±0.8 a	1.4±0.6 b
3	120 (5)	12	51.1±15.3 bc	39.8±4.3 c	78.4±8.1 d	18.0±3.1 e	7.4±2.3 c	1.1±0.2 a
4	297 (11)	20	52.0±14.1 c	46.2±5.2 b	93.1±9.6 b	20.6±2.9 c	7.2±2.1 c	1.2±0.4 a
5	384 (15)	29	49.7±10.6 bc	45.8±4.6 b	87.0±7.5 c	19.2±2.8 d	6.5±1.5 b	1.4±0.7 b
*F*			14.94*	50.59*	105.43*	79.39*	26.37*	18.37*

“*n*” is the total number of times the bat was LI, restricting analysis to three calls per call type. Same letter (a, b, c, d, e) indicates not significantly different from each other (Tukey multiple-comparison test). *: *P*<0.001.

Function 1 was most correlated with maximum frequency and function 2 was most correlated with duration. Large circles enclose areas with 75% probability of including calls from each bat.

### Call variants and cluster success

**Figure 7:**
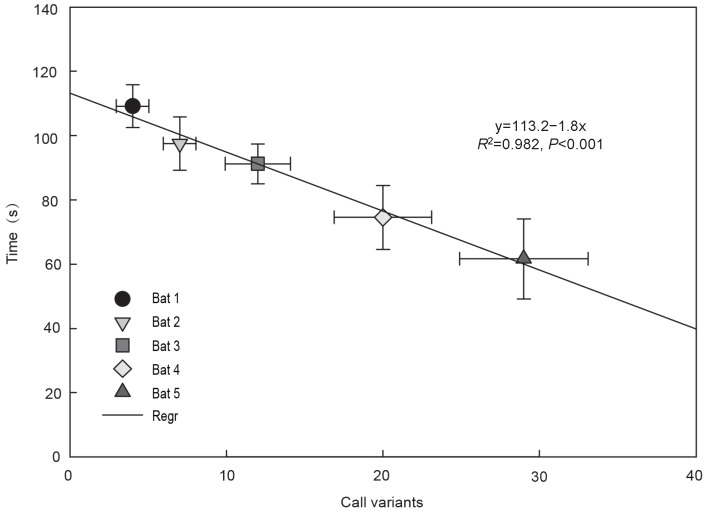
Relationship between call variants and time taken to join a cluster in *Myotis macrodactylus*

The clustering success of females was measured as the time taken to join a cluster (CT). We correlated the CT with the number of call variants emitted during one complete process of returning to the colony. Because call variants were most influenced by type and number of syllables, a negative correlation between the call variants of the LI and cluster success was derived (Figure 7). Clustering success tended to increase with the total number of call variants produced. The number of social calls for the five individuals varied from 4 to 29 during the entire research session. We found that call diversity was positively correlated with clustering efficiency. 

Solid line depicts linear regression.

## DISCUSSION

Intra-species variability of LI calls was found in the number of syllables, syllable order and syllable repetition across call renditions. These data confirmed that differences in social calls existed between *M*. *macrodactylus* individuals during the process of approaching the colony. Our results further confirmed the hypothesis that social call richness was significantly negatively correlated with time taken for the successful reunion of *M*. *macrodactylus* females in a cluster.

The LI can emit 10 simple monosyllabic and 27 complex multisyllabic call types composed of four syllable types. In previous studies, simple monosyllabic calls have been recorded in *Myotis* species at maternity roosts before bats leave and after they arrive (Pfalzer & Kusch, 2003), and usually begin with downward frequency-modulated signals. In mammals, repeated calls in communication signals can indicate a general coding rule (Fischer et al., 2001; Schehka et al., 2007). However, complex multisyllabic calls in *Pipistrellus pipistrellus* constitute different syllables, which may attract conspecifics and thereby help to deter predators (Budenz et al., 2009). We found that complex multisyllabic calls were emitted by *M*. *macrodactylus* females returning to the colony; furthermore, the diversity of calls was affected by syllable type and unique syllable types led to specific calls. New evidence has suggested that *Megaderma lyra* can regularly modify the structure of their calls, including the number and repetition of syllables, according to competing strength (Bastian & Schmidt, 2008), and signalers may convey their own properties in acoustic signals (Janßen and Schmidt, 2009). 

The sequences of pulses in *M*. *macrodactylus* female calls demonstrated a stable pattern. The social calls always began with a downward frequency-modulated component and were followed with one or two other sub-syllables; by adding syllables or types of syllables based on this rule, call variants were increased. Similar descriptions of calls with high diversity have been found in studies on *Saccopteryx bilineata* (Behr & von Helversen, 2004), *Tadarida brasiliensis* (Bohn et al., 2009) and *Pipistrellus nathusii* (Russ and Racey, 2007; Jahelková et al., 2008) courtship songs. These studies noted large inter-individual differences in calls, with males able to emit diverse courtship calls, and differences in individual calls most evident in regard to maximum frequency and duration. Higher diversity in the community may contribute to species identification in bats. In our study, individuals emitting low diversity calls required more time to join the group, and occasionally could not attain the central position. The border temperature of a group is lower than that of the central position, so bats need more energy consumption for thermogenesis. Therefore, the LI must repeatedly communicate with others when returning to the colony to obtain maximum benefit. To reduce the time taken for the recognition process, social behavior may drive the diversity of individual calls. 

We also found that the same calls were emitted by different individuals; these calls may function as identification signals among members of a colony and have a universal meaning. However, variation in the number and type of syllables may be due to specific information conveyed by individuals. Huddling together in a roost is likely a thermoregulatory strategy used by *M*. *macrodactylus* to conserve energy and increase survival or fitness. In this study, five individuals were randomly selected from 90 females, with no significant differences in forearm length or body size impacting the results. Each of the five bats could potentially be the LI, but the time spent alone by each bat was negatively correlated to their call diversity. Under natural circumstances, calls may be relevant to status identification. The clustering of numerous individuals would make diverse calls more identifiable by others. In this study, individuals that emitted multiple social calls may be more easily identified and accepted by others, such that individual calls may evolve towards complexity under natural selection. This article suggests a role for the sophisticated acoustic communication ability developed by bats. Further research is needed to elucidate the process of identification through social calls, which can be conducted through multiple methods and is affected by behavioral context. 

Our results indicated that the social calls emitted by clustering *M*. *macrodactylus* females always exhibited a stable structure and diversity. Furthermore, differences in social call variability between *M*. *macrodactylus* females was found, and individuals emitting more complex social calls took less time to rejoin the cluster. However, further work is needed to investigate the specific function of this diversity in social calls.
